# The influence of thionamides on intra-thyroidal uptake of ^131^I during radioiodine-131 treatment of Graves’ disease

**DOI:** 10.1038/s41598-023-47228-z

**Published:** 2023-12-01

**Authors:** Christian Happel, Benjamin Bockisch, Britta Leonhäuser, Amir Sabet, Frank Grünwald, Daniel Groener

**Affiliations:** Department of Nuclear Medicine, University Hospital, Goethe University, Theodor Stern Kai 7, D-60590 Frankfurt/Main, Germany

**Keywords:** Medical research, Thyroid diseases

## Abstract

Graves’ disease is one of the most common causes of hyperthyroidism. Guideline recommendations advocate the intake of thionamides for at least 1 year. If hyperthyroidism persists, subsequent radioiodine-131 treatment (RIT) is a therapeutic option. Thionamides are known to influence intra-thyroidal bio-kinetics of iodine and should therefore be discontinued at least 3 days prior to RIT if possible. However, the required therapeutic activity has to be calculated individually by pre-therapeutic measurement of the uptake prior to RIT [radioiodine-131 uptake test (RIUT)] in Germany according to national guidelines. Therefore, the aim of this study was to quantify the influence of thionamides on intra-therapeutic uptake. A cohort of 829 patients with Graves’ disease undergoing RIUT and RIT was analysed. Patients were subdivided into three groups. Group A: patients with carbimazole medication (n = 312), group B: patients with methimazole medication (n = 252) and group C: patients without thionamides (n = 265). Group A and B were further subdivided depending on the reduction of dosage of thionamides. In order to analyse the influence of thionamides, the variance of the determined individual extrapolated maximum intra-thyroidal uptake (EMU) between RIUT and RIT within the single groups and within the subgroups was statistically evaluated. When administering an equal dose of thionamides or no thionamides in RIUT and RIT (groups A1, B1 and C) no significant differences were detected when comparing EMU in RIT to EMU in RIUT (*p* > 0.05). In the subgroups A2–A4 (reduced dosage of carbimazole prior to RIT) EMU was significantly increased in RIT compared to RIUT [21% for a reduction of 0 to < 10 mg/d (A2), 39% for a reduction of 10–15 mg/d (A3) and 80% for a reduction of > 15 mg/d (A4)]. In the subgroups B2–B4 (reduced dosage of methimazole prior to RIT) EMU was as well significantly increased in RIT compared to RIUT [26% for a reduction of 0 to < 10 mg/d (B2), 36% for a reduction of 10–15 mg/d (B3) and 59% for a reduction of > 15 mg/d (B4)]. A significant dose-dependent increase of EMU in RIT compared to EMU in RIUT in patients discontinuing or reducing thionamides was detected. Therefore, thionamides should be discontinued at least 2 days prior to RIUT in order to achieve the designated target dose more precisely and to minimize radiation exposure of organs at risk.

## Introduction

The prevalence of benign thyroid diseases is constantly high^1–3^. Radioiodine-131 treatment (RIT) is a well-established non-invasive therapeutic option for patients suffering from hyperthyroidism^1,2,4,5^. For patients with Graves’ disease, RIT is applied to ablate target tissue related to autoimmunity and to reduce thyroid volume ^2,4,6,7^. Graves’ disease is known as an autoimmune disorder of the thyroid in which thyrotropin receptor stimulating antibodies (TRAb) permanently stimulate thyroid cells. This leads to an increase of synthesis and release of large amounts of thyroid hormones resulting in a clinical manifestation of thyrotoxicosis^8–10^.

The therapeutic approach to Graves’ disease initially consists of an inhibition of thyroid hormone synthesis by thyrostatic medication^3,8,11,12^. Methimazole and the methimazole analogue carbimazole are commonly used for anti-thyroid treatment^13–15^. Those drugs, also known as thionamides, are simple molecules containing a sulfhydryl group and a thiourea moiety within a heterocyclic structure^13–15^. Thionamides are actively concentrated in the thyroid against a concentration gradient and inhibit the synthesis of thyroid hormones by interfering with thyroid peroxidase-mediated iodination of tyrosine residues in thyroglobulin^15–19^. The intake of thionamides is generally well tolerated, but can cause significant adverse events, such as agranulocytosis, liver dysfunction and acute pancreatitis, when taken over longer periods of time^15^. Therefore, the duration of therapeutic intake of thionamides should usually be limited to 1 year. In approximately 50% of all patients, thyroid cells are able to produce a normal amount of thyroid hormone after the treatment^13,15–17^.

8, In cases where hyperthyroidism persists despite anti-thyroid treatment, RIT is a definitive non-invasive therapeutic option with few side effects compared to thyroid surgery^3,12,17–21^. Orally administered radioiodine-131 is selectively concentrated in thyroid cells and decays by emission of beta radiation with a physical half-life of 8 days and a mean β^−^ energy of 190 keV^22,23^. The emitted electrons lead to ionisation resulting in DNA damage^4,21,24,25^. The aim of RIT is a euthyroid state with or without the necessity of substitutional hormone medication^26^.

However, the achieved intra-thyroidal radiation dose depends on intra-therapeutic intra-thyroidal uptake of radioiodine-131, which is in turn strongly individual due to age, sex, additional medication, general condition and co-existing diseases^4,21,25,26^. Therefore, radioiodine-131 uptake testing (RIUT) prior to RIT is mandatory to estimate individual intra-thyroidal uptake and effective half-life for each individual patient^3,11,12,21,27^. In order to increase intra-therapeutic uptake of radioiodine-131, and therefore achieve higher radiation doses to the target tissue, national and international guidelines recommend a discontinuation of thionamides at least 2 days prior to RIT^3,12,26,28,29^. Therefore, RIUT is often performed 1 week prior to RIT under the influence of thionamides, while RIT itself is subsequently carried out after discontinuation of anti-thyroid drugs. However, a discontinuation of thionamides between RIUT and RIT may significantly alter intra-therapeutic intra-thyroidal uptake of radioiodine-131, and may therefore lead to an excess of the administered radiation dose to the target tissue^16^. When quantified absolutely and already considered in the pre-therapeutic calculation, the reduced required activity leads to a more exact target dose and a reduced radiation exposure of organs at risk and healthy tissue.

Aim of this study was therefore to evaluate the influence of thionamides on intra-thyroidal uptake in RIUT versus RIT in a retrospective monocentric analysis of a large cohort of patients suffering from Graves’ disease.

## Material and methods

In a retrospective monocentric study, a total of 1,167 patients suffering from Graves’ disease, who underwent both RIUT and RIT between 1999 and 2019, were evaluated. The study was approved by the local ethic committee (ethic committee of the Goethe University Frankfurt; 20-777). Inclusion criteria were either a constant dose of thionamides during RIUT and RIT or a reduced dose of thionamides between RIUT and RIT. An additional cohort of patients without any thyrostatic medication during RIUT and RIT served as control group. Exclusion criterion was a pre-therapeutically calculated effective intra-thyroidal half-life below 2 or beyond 8 days (133 patients). Furthermore, patients who did not receive a complete RIUT with measurements of the remaining activity 48 h and 96 h after administration of the RIUT activity were excluded (61 patients). A further exclusion criterion was a different anti-thyroid medication such as propylthiouracile or an insufficiently documented dosage of thionamides (n = 144).

Finally, a cohort of 829 patients (651 females, mean age: 52 ± 15 years) was included. These patients were subsequently subdivided into three different groups. Group A (carbimazole cohort) consisting of 312 Patients (244 females, mean age: 53 ± 14 years) with carbimazole medication during RIUT and RIT. Group B (methimazole cohort) consisting of 252 patients (201 females, mean age: 51 ± 16 years) with methimazole medication during RIUT and RIT. An additional control group (group C, no medication cohort) consisting of 265 patients (206 females, mean age: 52 ± 16 years) who did not receive any thionamides over a period of at least 1 week prior to RIUT and the complete duration of RIT. The groups A and B were further subdivided depending on the dosage of thionamides. The subgroups of group A were: A.1: No changes of carbimazole dosage between RIUT and RIT (n = 143), A.2: reduction of the carbimazole dosage between RIUT and RIT of less than 10 mg/d (n = 96), A.3: reduction of the dosage of carbimazole between RIUT and RIT of 10 to 15 mg/d (n = 54) and A.4: reduction of the dosage of carbimazole between RIUT and RIT of more than 15 mg/d (n = 19). Consequently, the subgroups for group B were defined as B.1: No changes of the dosage of methimazole medication between RIUT and RIT (n = 121), B.2: reduction of the methimazole dosage between RIUT and RIT of less than 10 mg/d (n = 92), B.3: reduction of the dosage of methimazole between RIUT and RIT of 10–15 mg/d (n = 30) and B.4: reduction of methimazole dosage between RIUT and RIT of more than 15 mg/d (n = 9). The majority of the investigated patients discontinued medication with thionamides completely (116/169 (70%) in the carbimazole group and 109/131 (83%) in the methimazole group). A reduction of thionamides was performed in patients in whom low dose maintenance was clinically necessary, e.g. in cases of relevant cardiac comorbidity, a history of atrial fibrillation and episodes of pronounced symptoms of thyrotoxicosis after complete discontinuation of thionamides. The demographic data of the final study cohort are shown in Table 1.

**Table 1:** Comparison of the investigated groups and subgroups regarding demographic and biokinetic data.

Group/subgroup	n	Female (%)	Male (%)	Age [a]	Thyroid volume [ml]	TSH [mU/l] RIUT (n)	Administered activity [MBq]	EMU RIUT Mean (95% CI)	EMU RIT Mean (95% CI)	EMU RIT/RIUT Mean (95%CI)	Target dose [Gy]
Complete cohort	829	79	21	52 ± 15	23 ± 14	0.70 ± 1.48 (533)	631 ± 296	0.47 (0.45–0.48)	0.49 (0.45–0.48)	1.14 (1.10–1.17)	274 ± 120
A: Carbimazole	312	78	22	53 ± 14	26 ± 17	0.87 ± 1.92 (153)	707 ± 298	0.45 (0.43–0.46)	0.49 (0.48–0.51)	1.21 (1.15–1.28)	270 ± 126
A1: Equal dosage	143	78	22	52 ± 14	26 ± 19	0.29 ± 0.57 (66)	708 ± 307	0.45 (0.42–0.48)	0.45 (0.42–0.47)	1.07 (0.99–1.15)	236 ± 122
A2: Reduction < 10 mg/d	96	84	16	54 ± 14	22 ± 14	1.28 ± 2.72 (50)	644 ± 284	0.45 (0.42–0.48)	0.50 (0.48–0.53)	1.21 (1.13–1.30)	296 ± 114
A3: Reduction 10–15 mg/d	54	70	30	52 ± 15	30 ± 14	1.10 ± 1.65 (28)	746 ± 284	0.46 (0.42–0.51)	0.57 (0.53–0.60)	1.39 (1.23–1.56)	282 ± 118
A4: Reduction > 15 mg/d	19	68	32	51 ± 12	29 ± 14	2.17 ± 2.38 (9)	899 ± 233	0.36 (0.29–0.43)	0.55 (0.47–0.63)	1.80 (1.25–2.37)	364 ± 141
B: Methimazole	252	80	20	51 ± 16	24 ± 14	0.82 ± 1.48 (187)	644 ± 305	0.48 (0.46–0.50)	0.52 (0.50–0.54)	1.19 (1.13–1.25)	288 ± 129
B1: Equal dosage	121	83	17	53 ± 17	26 ± 15	0.42 ± 0 .93 (90)	683 ± 503	0.51 (0.48–0.53)	0.50 (0.47–0.52)	1.06 (0.99–1.13)	250 ± 117
B2: Reduction < 10 mg/d	92	73	27	49 ± 15	22 ± 13	0.97 ± 1.32 (69)	590 ± 297	0.47 (0.43–0.50)	0.54 (0.51–0.56)	1.26 (1.17–1.36)	317 ± 106
B3: Reduction 10–15 mg/d	30	87	13	46 ± 14	25 ± 12	2.00 ± 2.66 (20)	630 ± 326	0.47 (0.40–0.53)	0.56 (0.50–0.62)	1.36 (1.13–1.59)	302 ± 141
B4: Reduction > 15 mg/d	9	78	22	56 ± 15	24 ± 16	1.02 ± 1.73 (8)	731 ± 222	0.37 (0.26–0.49)	0.56 (0.47–0.65)	1.63 (1.23–2.06)	450 ± 222
C: No mediacation	265	78	22	52 ± 16	19 ± 11	0.41 ± 0.77 (193)	530 ± 253	0.47 (0.45–0.49)	0.46 (0.44–0.47)	0.99 (0.96–1.01)	265 ± 103

Values indicated as mean ± standard deviation or 95% CI.

RIUT and RIT were performed in accordance with the German guidelines for RIUT and RIT in their current versions^11,12^. One week prior to inpatient RIT, RIUT was performed to determine the individual extrapolated-maximum-^131^I-uptake (EMU) and the intra-thyroidal effective half-life (EHL). Measurement of the remaining intra-thyroidal activity was performed with an individually calibrated gamma scintillation probe with connected multichannel analyser 48 h and 96 h after oral administration of the radioiodine-131 test capsule. EHL was estimated by mono-exponential fitting of the time activity curve. EMU was calculated by extrapolating the exponential fit to the intersection of the ordinate at time point zero. Thyroid volume was determined via ultrasound by experienced nuclear medicine physicians. The mass of the thyroid, that is necessary for dosimetrical estimation, was calculated using the determined volume and a postulated density of the thyroid tissue of 1 g/ml^21^.

The designated target dose to the thyroid was 250 Gy for initial RIT and 300 Gy in cases of subsequent RIT after initial treatment failure^12^. The required activity was calculated by experienced medical physicists using the Marinelli-equation^3,11,30^. According to current German guidelines, patients were hospitalized for at least 48 h after oral administration of the radioiodine-131 capsule^11,12,25^. Patients were discharged after falling below the German regulatory limit of 250 MBq for residual activity in the body^11,12^. During in-patient stay, a measurement of the remaining intra-thyroidal activity was performed twice a day using the same model of a calibrated gamma scintillation probe with connected multichannel analyser as used for RIUT. Intra-therapeutic EHL was again calculated using a mono-exponential fit of the time activity curve. EMU was again calculated by extrapolating the aforementioned exponential fit to the intersection of the ordinate at time point zero.

In order to analyse the influence of anti-thyroid medication on individual intra-thyroidal uptake the variance of the determined individual EMU between RIUT and RIT within the single main groups and within the subgroups as well as the variance of the individual EMU in comparison to the subgroups was evaluated statistically. Statistical evaluation was performed using analysis of variance (ANOVA) for comparison of the different cohorts. The 95% confidence interval of the mean was calculated for each subgroup. For evaluation of intra-individual changes paired t-test was used. Linear regression was performed to evaluate significance of the dose-dependence of changes in the EMU. Statistical significance was indicated with *p*-values < 0.05.

### Ethics approval

All procedures performed in studies involving human participants were in accordance with the ethical standards of the institutional and/or national research committee and with the 1964 Helsinki declaration and its later amendments or comparable ethical standards. This article does not contain any studies with animals performed by any of the authors. Informed consent Informed consent was obtained from all individual participants included in the study.

## Results

**Figure 1:**
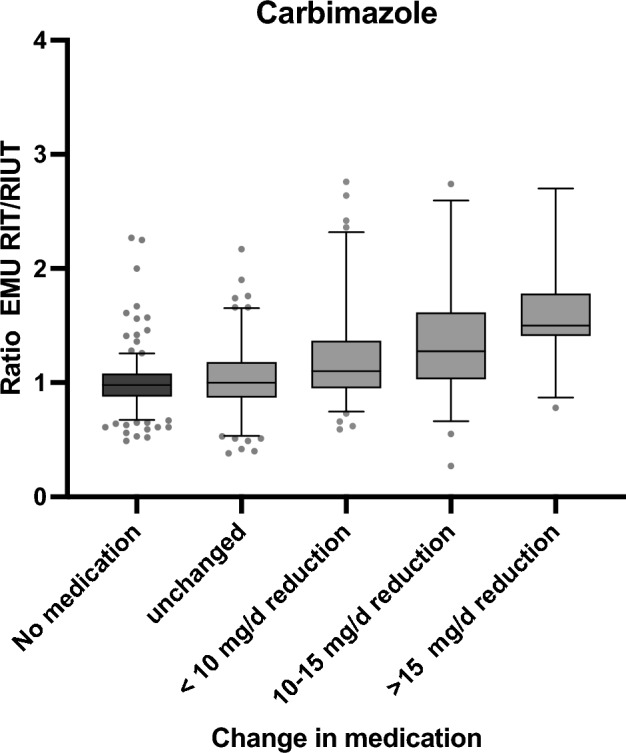
Comparison of the relative EMU increase depending on the reduction of carimazole dosage between RIUT and RIT.

**Figure 2:**
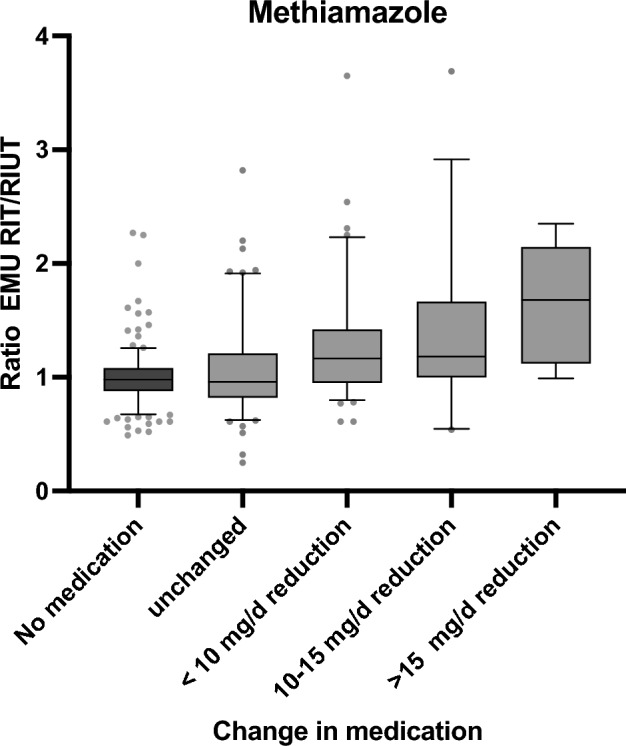
Comparison of the relative EMU increase depending on the reduction of methimazole dosage between RIUT and RIT.

No significant differences could be detected between the groups. The analysis of group A (carbimazole) and group B (methimazole) revealed a statistically significant increase of the intra-therapeutic EMU compared to EMU in RIUT. In group A (carbimazole), EMU increased from 0.45 (95% CI 0.43–0.46) in RIUT to 0.49 (95% CI 0.48–0.51) in RIT (+ 10%) (*p* < 0.05). In group B (methimazole), EMU increased from 0.48 (95% CI 0.46–0.50) in RIUT to 0.52 (95% CI 0.50–0.54) in RIT (+ 8%) (*p* < 0.05). For group C (no thyrostatic medication), mean EMU was 0.47 (95% CI 0.45–0.49) in RIUT and 0.46 (95% CI 0.44–0.47) in RIT. This slight decrease was not statistically significant (*p* > 0.05). The determined data for the complete cohort and the subgroups are summarized in Table 1. The comparison of the increase of EMU between RIUT and RIT within the different groups and subgroups is shown in Figs. 1 and 2. The increase of EMU in group A (carbimazole) and group B (methimazole) showed a highly significant difference compared to group C (no thionamides) (*p* < 0.05).

The comparison of the subgroups A1–A4 revealed a significant association of the increase of EMU in RIUT versus EMU in RIT and higher levels of thionamide dose reduction (*p* < 0.05). Mean increase of EMU between RIUT and RIT was 21% (95% CI 13–30%) for a reduction of 0–< 10 mg/d carbimazole (A2), 39% (95% CI 23–56%) for a reduction of 10–15 mg/d carbimazole (A3) and 80% (95% CI 25–137%) for a reduction of > 15 mg/d carbimazole (A4) (Table 1, Fig. 1). In patients receiving an equal dosage of carbimazole in RIUT as well as in RIT (A1), the mean EMU remained stable with an EMU of 0.45 (95% CI 0.42–0.48) in RIUT and 0.45 (95% CI 0.42–0.47) in RIT (*p* > 0.05).

The comparison of the subgroups B1–B4 revealed a highly significant association of the increase of EMU between RIUT and RIT with higher levels of thionamide dose reduction (*p* < 0.05), as well. Mean increase of EMU between RIUT and RIT was 26% (95% CI 17–36%) for a reduction of 0–< 10 mg/d methimazole (B2), 36% (95% CI 13–59%) for a reduction of 10–15 mg/d methimazole (B3) and 63% (95% CI 23–106%) for a reduction of > 15 mg/d methimazole (B4) (Table 1, Fig. 2). In patients receiving an equal dosage of methimazole in RIUT as well as in RIT (B1), the mean EMU remained stable with 0.51 (95% CI 0.48–0.53) in RIUT and 0.50 (95% CI 0.47–0.52) in RIT (*p* > 0.05). Table 1 and Figs. 1 and 2 show the mean ratio of EMU in RIT and EMU in RIUT, and therefore the relative increase of EMU between RIUT and RIT (Fig. 1; Fig. 2).

## Discussion

Radioiodine-131 therapy is a well-established therapeutic option in Graves’ disease. However, metabolic distribution and secretion of radioiodine-131 significantly depends on several influencing factors such as previous administration of radioiodine-131 or additionally administered drugs^5,25^. Therefore, the exact calculation of the required activity, that leads to an optimum target dose is controversially discussed^17,24,31–34^. The presented study aims to investigate the influence of thionamides on intra-thyroidal radioiodine-131 uptake in RIT in a large cohort of patients suffering from Graves’ disease.

Iodine specifically accumulates in thyroid follicles^35,36^. The extent of intra-thyroidal iodine uptake is mediated by TSH and strongly dependant on the activity of sodium-iodine-symporters on the thyroid cell surface^21,25,36^. However, in patients with a low intra-thyroidal iodine uptake undergoing a RIT, the required activity of radioiodine-131 has to be extended to receive the targeted intra-thyroidal radiation dose of 250 Gy. National and international guidelines prescribe a discontinuation of thionamides several days prior to therapeutic administration of radioiodine-131^3,12^. By discontinuation of thionamides, intra-thyroidal uptake can be increased causing a decrease of the administered activity of radioiodine-131 and therefore a reduced radiation dose to organs at risk. However, the magnitude of the dose dependence is currently unknown. Therefore, only an absolute quantification of this effect and a consideration of these results already during the calculation of the required therapeutic activity of radioiodine-131 will lead to an adequately performed RIT. In the presented study, the intra-therapeutic EMU compared to pre-therapeutic EMU showed a highly significant increase with rising levels of thionamide dose reduction.

The influence of thionamides on the success of RIT is well documented. Sabri et al. investigated 207 patients with Graves’ disease depending on the intake of thionamides during RIT. The success rate of RIT was 49% in patients who took thionamides but 93% in patients without thionamides. The authors concluded that thionamides should be discontinued at least 1 day prior to RIT to avoid a negative influence on bio-kinetics of radioiodine-131 during RIT^29^. Zantut-Wittmann et al. likewise found a higher risk of treatment failure in patients who took thionamides during RIT compared to those patients who discontinued thionamides prior to treatment. The authors concluded that this effect was caused by a reduced intra-thyroidal uptake^17^. Urbannek et al. compared 385 patients with Graves’ disease who underwent a RIT. The authors subdivided their cohort in a group of patients who discontinued thionamides 2 days prior to RIT, and a group of patients who continued thionamides during RIT. The authors found a significantly higher intra-thyroidal uptake in the group of patients who discontinued thionamides^37^. Andrade et al. investigated a cohort of 61 patients in a prospective randomized study and did not find a difference in outcome of RIT with and without thyrostatic medication. However, thionamides were discontinued at least 4 days prior to RIT^38^. Sundawa et al. recommended to discontinue thionamides for at least 3 days prior to RIT. The authors investigated a cohort of 39 patients and found a significantly higher therapeutic success rate when withdrawing thionamides at least 3 days prior to RIT^28^. Hancock et al. postulated a radio protective effect of thionamides and recommended an empirical increase of administered radioiodine-131 in patients who recently received thionamides. The authors concluded, that a discontinuation of thionamides is associated with a rapid rise in thyroid hormone levels within 2–3 days of discontinuation^10^.

The aforementioned studies clearly demonstrate the importance of discontinuing thionamides prior to RIT due to the influence on intra-thyroidal uptake of radioiodine-131. However, thionamides were discontinued shortly before RIT, while RIUT was performed under the influence of thionamides. Kobe et al. included 571 Patients with Graves’ disease undergoing a RIT and investigated a follow up of 12 months. The patients in their cohort discontinued thionamides even prior to RIUT. The authors found a success rate of 96% and concluded, that thionamides should be discontinued at least 2 days before RIUT to eliminate hyperthyroidism^35^.

In the presented study, the cohort of patients receiving thionamides was subdivided in patients receiving carbimazole (group A) and patients receiving methimazole (group B). These groups were further subdivided depending on the reduction of thionamides between RIUT and RIT. The subgroups were defined as a reduction of less than 10 mg/d (A/B 2), a reduction of 10–15 mg/d (A/B 3) and a reduction of the dosage of thionamides of more than 15 mg/d (A/B 4). Methimazole is the active metabolite of carbimazole^14^. Carbimazole is converted to methimazole in vivo and equivalent doses of carbimazole and methimazole are supposed to be 0.6–1.0^39^. Therefore, a direct comparison of carbimazole and methimazole is not meaningful. However, the dose-dependant increase of EMU was significant in both groups Fig. 1, 2.

Another important aspect controversially discussed is the duration of the pre-therapeutic RIUT. Bogner et al.^32^ caution that a RIUT of 5 days may be too short and potentially lead to an intra-individual variability of radioiodine bio-kinetics. In the presented study a RIUT with measurements of the remaining intra-thyroidal activity 48 and 96 h after administration of the test capsule was performed. A late measurement of the remaining intra-thyroidal activity (for example 168 h after administration) was not possible due to organisation reasons^25^.

## Conclusion

The presented results show a significant dose-dependent increase of the intra-therapeutic EMU compared to pre-therapeutic EMU in patients with Graves’ disease discontinuing thionamides prior to RIT. The influence of thionamide dosage change on intra-thyroidal uptake of radioiodine-131 was quantified for the first time. Thionamides should be discontinued at least 2 days prior to RIUT. If this is not possible, the investigated effect should be considered when calculating the required activity for RIT. Hereby, the scheduled target dose will be achieved more precisely and the radiation exposure of organs at risk should be minimized.

## Data Availability

The datasets generated during and/or analysed during the current study are available from the corresponding author on reasonable request.
